# 

**Published:** 1983-04

**Authors:** 


### Centenary Year
### 1883–1983

### BRISTOL MEDICO-CHIRURGICAL JOURNAL

APRIL 1983
VOLUME 98 (ii)
NUMBER 366

